# Evaluation of Chromosomal Instability in Diabetic Rats Treated with Naringin

**DOI:** 10.1155/2011/365292

**Published:** 2011-09-20

**Authors:** Saleh A. Bakheet, Sabry M. Attia

**Affiliations:** ^1^Department of Pharmacology and Toxicology, College of Pharmacy, King Saud University, P.O. 2457, Riyadh 11451, Saudi Arabia; ^2^Department of Pharmacology and Toxicology, College of Pharmacy, Al-Azhar University, Cairo, Egypt

## Abstract

We used the bone marrow DNA strand breaks, micronucleus formations, spermatocyte chromosomal aberrations, and sperm characteristic assays to investigate the chromosomal instability in somatic and germinal cells of diabetic rats treated with multiple doses of naringin. The obtained results revealed that naringin was neither cytotoxic nor genotoxic for the rats at all tested doses. Moreover, naringin significantly reduced the diabetes-induced chromosomal instability in somatic and germinal cells in a dose-dependent manner. In addition, diabetes induced marked biochemical alterations characteristic of oxidative stress including enhanced lipid peroxidation, accumulation of oxidized glutathione, reduction in reduced glutathione, and accumulation of intracellular reactive oxygen species. Treatment with naringin ameliorated these biochemical markers dose-dependently. In conclusion, naringin confers an appealing protective effect against diabetes-induced chromosomal instability towards rat somatic and germinal cells which might be explained partially via diminishing the *de novo* free radical generation induced by hyperglycemia. Thus, naringin might be a good candidate to reduce genotoxic risk associated with hyperglycemia and may provide decreases in the development of secondary malignancy and abnormal reproductive outcomes risks, which seems especially important for diabetic patients.

## 1. Introduction

Diabetes mellitus is a serious health problem affecting millions of individuals worldwide. By the year 2025, the World Health Organization (WHO) predicts that 300 million people will have diabetes mellitus [1]. Oxidative stress is thought to play a significant role in the aetiopathology of a wide variety of diseases such as chronic inflammation, atherosclerosis, and cancer [2]. It can be also associated with type II diabetes mellitus and reactive oxygen species produced during this stress may cause DNA damage [3, 4]. Moreover, individuals with diabetes mellitus have reduced antioxidant defence capacity [5]. DNA damage and repair play a major role in neoplastic transformation, because mutations in DNA repair genes can be directly related with cancer and the efficacy of DNA repair may determine the susceptibility to carcinogenesis [6]. The oxidative damage to the germinal cells is considered to be the leading cause for the various reproductive related complications resulting in infertility and other congenital and developmental defects [7]. It has been demonstrated that diabetes mellitus is associated with elevated level of oxidative DNA damage and with the increased susceptibility to mutagens and the decreased efficacy of DNA repair [8, 9]. This can contribute to the chromosomal instability in diabetics and, in consequence, to cancer [10]. Epidemiological data suggest a positive correlation between diabetes mellitus and increased cancer risk, but the mechanism underlying this correlation and precise relationship between chronic hyperglycemia and cancer are still obscure [11].

Treatment of diabetes mellitus and its complications in the recent context has focused on the usage of plant extracts and their constituents. The WHO had estimated that approximately 80% of the Earth's inhabitants rely on traditional medicine for their primary health care needs, and most of this therapy involves the use of plant extracts or their active components [12]. Grapefruit is part of the diet in most countries, where it is often consumed regularly as juice. The chemical responsible for the characteristic sour flavour of the fruit is naringin, a flavonone that is rapidly transformed into naringenin by the action of the enzymes *α*-ramnosidase and *β*-glucosidase [13]. Naringin exhibits various pharmacological and therapeutic properties including action as anticancer, anti-inflammatory, and cardioprotective activity [14]. Moreover, naringin decreased blood glucose levels and increased plasma insulin levels in streptozotocin-induced diabetic rats [15, 16]. Like most flavonoids, naringin has metal chelating, antioxidant, and free radical scavenging properties and has been reported to offer some protection against lipid peroxidation [17, 18]. 

Much interest has surrounded the role and use of natural antioxidants as a mean of preventing oxidative damage in diabetes mellitus with high oxidative stress. In this context, our aim was to evaluate the potential antigenotoxic utility of naringin against diabetes-induced chromosomal instability towards rat somatic and germinal cells and to elucidate the possible mechanism whereby naringin mediates its beneficial effects. Collecting these data might lead to focusing on development of novel antigenotoxic agents that are commonly consumed in human diet. In laboratory animals, streptozotocin and alloxan show selective cytoxicity in pancreatic *β*-cells and thus cause both type I and type II diabetes mellitus. Diabetics and experimental animal models exhibit high oxidative stress due to persistent and chronic hyperglycemia, depleting the activity of antioxidant defence system and thus promoting *de novo* free radical generation [19]. Generated free radicals can attack all types of macromolecules including DNA causing chromosomal instability that may lead to mutagenesis or carcinogenesis [20]. The somatic cell chromosomal instability was assessed by the bone marrow comet assay and micronucleus test. Spermatocyte chromosomal analysis and spermiograms examination were undertaken in the current study as markers of germ cell chromosomal instability. In addition, oxidative stress markers such as sperm lipid peroxidation, oxidized glutathione (GSSG), reduced glutathione (GSH) levels, and reactive oxygen species (ROS) were assessed as a possible mechanism underlying this amelioration.

## 2. Results

### 2.1. Effect of Naringin on Diabetes-Induced DNA Strand Breaks

The results of alkaline comet assay are shown in Table 1. Naringin treatment did not exhibit any significant difference in the level of tail length, tail DNA, tail moment, and olive tail moment compared to the solvent control at either dose tested. The results revealed that diabetes causes significant increase in the level of all measured parameters in comparison to those of the solvent control group. However, when both doses of naringin were given to diabetic animals for 4 weeks, decreased rates of DNA strand breaks were significantly observed and the higher dose of naringin gave the more effective reduction in all measured parameters (*P* < 0.01).

### 2.2. Effect of Naringin on Diabetes-Induced MNPCE

The results of the MN test are presented in Table 2. Significant increase in the frequency of MNPCE was observed in diabetic rats compared to the solvent control group (*P* < 0.01). Naringin treatment did not exhibit any significant difference in the frequency of MNPCE compared to the solvent control at both tested doses. With regard to the diabetic animals treated with naringin, a weak protection was observed with 25 mg/kg of naringin (*P* < 0.05). With 50 mg/kg, however, naringin produced a clear significant inhibitory effect on the MNPCE induced during diabetes in comparison to the untreated diabetic rats (*P* < 0.01). The results for the rate of PCE : NCE are also presented in Table 2. It is shown that no significant decrease in the percent PCE was observed when the bone marrow cells were sampled from diabetic rats (*P* > 0.05). Similarly, none of the tested doses of naringin modified the ratio in comparison with the control group.

### 2.3. Effect of Naringin on Diabetes-Induced Spermatocyte Chromosomal Aberration

The results of the spermatocyte diakinesis-metaphase I analysis are shown in Table 3. Naringin treatment did not exhibit any significant difference in the frequency of structural or numerical (polyploides) chromosomal aberrations compared to the solvent control at both tested doses. The results revealed that diabetes induced aberrant primary spermatocytes, which are statistically significant (*P* < 0.05) compared with that of the nondiabetic control group. Nevertheless, at both doses tested, naringin treatment reduced the total frequency of aberrant primary spermatocytes in diabetic rats in comparison to untreated diabetic group, and higher dose of naringin gave more effective reduction in the frequency of total chromosomal aberrations (*P* < 0.01). Moreover, the administration of higher dose of naringin produced a response which was close to the one observed with the solvent control.

### 2.4. Effect of Naringin on Diabetes-Induced Alterations in Spermatozoa Count and Abnormalities

The results of the sperm cell experiments are shown in Table 4. Treatment of rats with naringin did not affect the parameters studied as compared to the nondiabetic control value at both tested doses. Sperm count was significantly decreased in untreated diabetic animals as compared to the value observed in the control group. Moreover, diabetes caused a significant increase in the percentage of abnormal sperm (*P* < 0.05). Treatment of rats with naringin for 4 weeks after diabetes induction increases the decreased percentage of sperm count. Moreover, the percentage of abnormal sperm was also restored with naringin treatment and higher dose of naringin gave more amelioration (*P* < 0.01).

### 2.5. Effect of Naringin on Diabetes-Induced Sperm Oxidative Stress

The effect of naringin on the diabetes-induced oxidative stress in rats was assessed by measuring sperm MDA content, GSH, GSSG levels, and ROS accumulation. The results of these experiments are also shown in Table 4. No significant change in MDA content was observed in sperm cells after naringin treatment compared to the control at both tested doses. The MDA content in diabetic animals was significantly increased as compared to control group (*P* < 0.05). Diabetes-induced MDA formation was abrogated by naringin at the two tested doses and decreased to level significantly different from the level of MDA in untreated diabetic animals. Higher dose of naringin gave more protection (*P* < 0.01). 

Sperm GSH and GSSG levels did not show any significant variation in naringin-treated animals compared to the control. The GSH level observed in diabetic animals was significantly decreased, together with increase in GSSG level as compared to the control group (*P* < 0.05), so that GSH/GSSG ratio significantly decreased, indicating increased oxidative stress (*P* < 0.05). Diabetic animals treated with both doses of naringin showed a significant increase in GSH level over the untreated diabetic animals and increased to level significantly different from the level of GSH in the untreated diabetic rats. The GSSG level was also significantly decreased in diabetic animals treated with both doses of naringin compared to untreated diabetic rats. Consequently, the GSH/GSSG ratio was increased in naringin treated diabetic animals and was statistically significant when compared to the untreated diabetic rats. The maximum increase in GSH/GSSG ratio was also observed in diabetic animals treated with naringin 50 mg/kg.

Sperm ROS production was evaluated by determining the fluorescent intensity of DCF. DCF fluorescence level did not show any significant variation after treatment of rats with both doses of naringin as compared to the control. The sperm DCF fluorescence level observed in diabetic animal was significantly increased by about 1.6-folds as compared to the control (*P* < 0.05). However, diabetes-induced production of DCF fluorescence was profoundly abrogated by both doses of naringin and decreased to the level significantly different from the level of DCF fluorescence in untreated diabetic animals (*P* < 0.01).

## 3. Discussion

Diabetes mellitus itself is not responsible for a high mortality and morbidity among diabetic patients. They are caused mainly by its complications, first of all coronary heart disease, which are, at least in part, consequences of oxidative stress associated with diabetes. Some reports suggest also that diabetes can be associated with cancer, but the mechanism underlying this association is unclear. A previous study demonstrated that diabetes was linked with the elevated level of oxidative DNA damage, the increased susceptibility to mutagens, and the decreased efficacy of DNA repair [8]. Because oxidative DNA damage may contribute to cancer promotion and progression, it can be considered as an element of the link between diabetes and cancer [9]. In this context, using natural antioxidative compounds would have a benefit in preventing diabetes oxidative stress-related consequences. Importantly, certain antioxidants are known to have genotoxic or carcinogenic potentials [21, 22]; however, the treatment with naringin, in the present study, was devoid of any genotoxicity and/or cytotoxicity in nondiabetic and diabetic rats. Moreover, treatment with naringin was found to protect against diabetes-induced increase of somatic and germinal chromosomal instability. These observations confirm earlier studies in which naringin was reported to prevent radiation or lomefloxacin-induced chromosomal instability in animal models [23, 24]. 

The genotoxic effects of the diabetogenic agent streptozotocin observed in the current study are in agreement with results obtained from previous animal studies [25, 26]. The observed chromosomal instability in the rats with experimental diabetes could be explained by many physiological changes caused by the pancreatic *β*-cell destruction induced by streptozotocin. This damage may trigger inflammatory processes, as well as hyperglycaemia-induced oxidative stress [27], that may be sufficient to increase the chromosomal damage. A prolonged diabetic state of rats before starting the sampling has produced high amounts of additional damage in the diabetic rats; this could be due to the metabolic alterations that occurred as a result of diabetes, as it happens in humans with long-term hyperglycaemia who acquire diverse vascular and renal dysfunctions. These pathologies may cause inflammatory reactions that also are associated with increased free radical production and, as a consequence, with increased DNA damage [28, 29]. In fact, elevated levels of DNA damage evaluated with the sister chromatid exchange, MN, and alkaline comet assay were reported in patients with type II diabetes mellitus [8, 30, 31]. It is becoming increasingly evident that an increased rate of DNA damage or chromosome breakage in somatic cells is an important risk factor for cancer [32].

Reproductive disturbances in diabetic males are well established and diabetes is the most common cause of erectile dysfunction in men. Moreover, poor semen quality has also been reported in diabetic men, including sperm concentration and increased abnormal sperm morphology [33]. In animal models of diabetes, induced by either streptozotocin or alloxan, earlier works have demonstrated various male reproductive dysfunctions both structurally and functionally [34, 35]. In the current study we have ascertained the germ cell genotoxicity in terms of incidence of spermatocyte chromosomal aberration, sperm count, and abnormal sperm morphology. Marked alteration in these studied parameters during the progressive phase of diabetes possibly confirms that the diabetes induction has a significant effect on spermatogenesis, sperm maturation, and development during storage and transit. Higher incidence of spermatocyte chromosomal aberration, reduced sperm counts and higher abnormal sperm during the progressive phase of diabetes may be interpreted as a combined effect of reduced leydig cell function (as evidenced by reduced testosterone levels in serum/testis) and diabetes-associated oxidative stress [36]. The current results confirm earlier studies in which elevated genotoxicity and oxidative stress were observed in the spermatozoa of diabetic rats [26, 37]. 

Induction and repair of DNA strand breaks and alkali-labile sites by streptozotocin was analyzed by several authors. Mossman et al. [38] showed that streptozotocin induces DNA single-strand breaks in a rat insulinoma cell line (RINr 38) and that these lesions can be repaired in a time-dependent manner, with most repair completed by 24 h post-exposure to streptozotocin. Using the same cell line, Pettepher et al. [39] demonstrated that streptozotocin induces alkali-labile sites in a dose-dependent fashion within the mitochondrial DNA and that these lesions can be repaired. Pettepher et al. [39] found that 8 h after exposure to the antibiotic, 55% of the lesions induced in the mitochondrial DNA were removed. The level of repair increased to 70% after 24 h. 

On the other hand, in our study the diabetic rats were euthanized approximately 4 weeks after the streptozotocin administration and the frequencies of chromosomal instability and oxidative stress markers were significantly higher in those diabetic rats as compared to the control. Thus, streptozotocin *per se* was not responsible for the increased levels of chromosomal instability in diabetic rats. This fact may be justified by Junod et al. [40] in an attempt to follow the metabolism of synthesized streptozotocin, (1-^14^C)-streptozotocin, (2-^14^C)-streptozotocin, and (3-methyl ^14^C)-streptozotocin (specific activity 1–2.7 *μ*Ci/mg) in rats. Firstly, the urine collected during the first 1 h drug contained the highest proportion of the injected radioactivity (34% with 3-methyl ^14^C)-streptozotocin, 40% each with (1-^14^C)-streptozotocin and (2-^14^C)-streptozotocin. This larger proportion suggested that either unchanged streptozotocin or its metabolites were rapidly eliminated from the body. Therefore, the increased levels of chromosomal instability in somatic and germinal cells in diabetic rats are likely due to the oxidative stress resulting from hyperglycemia. Hyperglycemia, a well-recognized pathogenetic factor of long-term complications in diabetes mellitus, not only generates more ROS but also attenuates antioxidative mechanisms through glycation of the scavenging enzymes [41].

The mechanism of inhibition of diabetes-induced chromosomal instability in the present study is not clearly understood. One possible explanation is that treatment with naringin would allow interception of free radicals generated by diabetes before they reach DNA. In the present work, in order to evaluate whether the observed antigenotoxic effect was due to an enhancement of the scavenger of free radicals generated by diabetes, oxidative stress markers such as lipid peroxidation, GSH/GSSG ratio and ROS accumulation, were evaluated 4 weeks after diabetes induction. The present study demonstrates that naringin reduced the diabetes-induced sperm lipid peroxidation and ROS accumulation and prevented the reduction in GSH/GSSG ratio significantly. The increased GSH level suggests that protection by naringin may be mediated through the modulation of cellular antioxidant levels. These observations confirm earlier studies in which naringin was reported to scavenge free radicals and lipid peroxides [15, 24]. Thus, scavenging of these free radicals by naringin seems to be an important mechanism against the hyperglycemia-induced chromosomal instability. 

The detailed mechanisms of the naringin antigenotoxic action remain to be investigated in future. In the current study, we tried to confirm one possible mechanism of naringin's antigenotoxic action, that is, inhibition of free radical generation. The results demonstrated that free radical generation by hyperglycemia was considerably inhibited by the treatment with naringin. In addition, analysis of gene expression for genes related to oxidative stress, measurements of more sensitive markers for lipid peroxidation, or oxidative DNA damage and immunocytochemical detection for these markers might be interesting and might provide molecular insights for the protective effect of naringin against chromosomal instability induced by hyperglycemia. However, at present diabetic patients who consume naringin as part of their diet should also consider its additional, beneficial effect—a possible decrease in chromosomal instability, which seems especially important for diabetic patients.

## 4. Materials and Methods

### 4.1. Animals

Adult Wistar albino male rats weighing 250–300 g (10–12 weeks old) were obtained from Experimental Animal Care Center, College of Pharmacy, King Saud University. The animals were maintained under standard conditions of humidity, temperature (20 ± 2°C), and light (12-h light/12-h dark). They were fed with a standard rats pellet diet and had free access to water. All animal experimentation described in the manuscript was conducted in accordance with accepted standards of humane animal care in accordance with the NIH guidelines and the legal requirements in Kingdom of Saudi Arabia.

### 4.2. Drugs

Naringin and streptozotocin were purchased from Sigma (Sigma-Aldrich St. Louis, MO, USA). Streptozotocin was dissolved in freshly prepared sodium citrate buffer (pH 4.4) while naringin was dissolved in distilled water and administered by gavage following preparation. Control animals were received equal amounts of distilled water. All other chemicals were of the finest analytical grade.

### 4.3. Induction of Diabetes in Rats

The overnight fasted rats were made diabetic by a single intraperitoneal injection of freshly prepared streptozotocin (65 mg/kg) in citrate buffer in a volume of 1 mL/kg. Three days after streptozotocin administration, the blood glucose level of each rat was determined. Rats with blood glucose levels of >350 mg/dL were considered diabetic and included in the study. Treatment with naringin was started on the third day after streptozotocin injection (i.e., after the estimation of blood glucose).

### 4.4. Experimental Design

Six groups of 5 adult rats were used. Three groups of them were first made diabetic as described above. When diabetes was confirmed, two groups were given 25 or 50 mg/kg naringin orally using an intragastric tube daily for a period of 4 weeks and the other two nondiabetic groups were also daily received by oral intragastric tube doses of 25 or 50 mg/kg naringin for 4 weeks. The doses of naringin were selected on the basis of its the antihyperglycemic and antioxidant effects in streptozotocin-induced diabetes in male Wistar rats [16]. Untreated nondiabetic and diabetic groups were also included in the experiment. The animals were killed by cervical dislocation under light anesthesia at week 4 post naringin treatments, then bone marrow cells, testes, and sperm cells were sampled. Naringin has been described to be present in grapefruit juice at about 101 mg/litre [42]. Upon conversion of animal dose to the equivalent human dose (human dose (mg/kg) = rat dose (mg/kg) × (6/37)), a dose of 50 mg/kg naringin in rats corresponded to 8.1 mg/kg in humans. Accordingly, for an average person weighing 60 kg, 486.5 mg naringin would be needed. Based on the previously mentioned concentration range, 486.5 mg naringin would be contained in 0.5 litre of grapefruit juice.

### 4.5. Detection of Bone Marrow DNA Strand Breaks

Rats were sacrificed by cervical dislocation, bone marrow cells from one femur were collected in tubes containing ice-cold PBS (Ca^2+^ and Mg^2+^ free, pH 7.4), and DNA strand breaks were studied by alkaline single cell gel electrophoresis (alkaline comet assay) according to the guidelines of Tice et al. [43] with slight modifications as previously described [44]. The slides were stained with ethidium bromide (20 *μ*g/mL) and studied using a fluorescent microscope (Nikon, Japan) equipped with appropriate filter. Fifty individual cells were selected for calculations for each analysis; all experiments were carried out at least three times, each with two parallel slides per animal. Single cells were analyzed with TriTek CometScore version 1.5 software. The parameters studied to access the DNA damage were the tail length (*μ*m), tail DNA (%), tail moment (arbitrary units), and olive tail moment (arbitrary units).

### 4.6. Bone Marrow Micronucleus Test

The remaining femur was used for estimation of micronuclei (MN) frequencies and mitotic activity. The bone marrow cells were collected in tubes containing foetal calf serum and centrifuged for 10 minutes at 1,100 rpm. The pellets were resuspended in a small volume of foetal calf serum for smear preparation. Two smears of bone marrow were prepared from each rat. After air drying, the smears were coded and stained by May-Gruenwald/Giemsa as described previously [45]. From each animal, 1,000 polychromatic erythrocytes (PCEs) and 1,000 normochromatic erythrocytes (NCEs) were examined for the presence of MN under 1,000x magnifications using a Nikon microscope. In addition the number of PCEs among 1,000 NCE per animal was recorded to evaluate bone marrow suppression, mitotic activity. The value was expressed as % PCEs of the total erythrocyte counts to determine the reduction of erythroblast proliferation.

### 4.7. Primary Spermatocyte Chromosomal Analysis

Immediately after cervical dislocation, both testes used were removed and placed in 2.2% isotonic tri-sodium citrate solution. The tunica albugenia was peeled out and somniferous tubules were teased to form a cell suspension. The suspension was centrifuged for 5 minutes at 1,000 rpm and the pellet was resuspended in 1.1% hypotonic tri-sodium citrate solution for 20 minutes at room temperature. After centrifugation the supernatant was discarded and the pellets were resuspended in Carnoy's fixative. Two to three changes of fixative were required before the preparation of slides. Finally, the cells were suspended in 1 mL of fixative and burst open on a clean slide to release chromosomes. At least four slides were made for each animal and allowed to dry overnight. The coded slides were stained with giemsa and scored as detailed earlier [46]. Hundred well-spread diakinesis-metaphase I cells per animal were analyzed under bright field microscope for chromosomal aberrations. The types of aberrations recorded in diakinesis-metaphase I cells included univalents, fragments, breaks, polyploids, and multivalents having a chain of four chromosomes. The percentage of total chromosomal aberrations for each group was calculated.

### 4.8. Cauda Epididymal Spermatozoa Evaluation

For sperm characteristic analysis, one epididymal content of each rat was collected to estimate the sperm abnormalities and sperm counts. Immediately after cervical dislocation, one caudae epididyme of each animal was dissected and incisions were made. Then the epididymes were placed individually into tubes filled with 3 mL of fetal calf serum. The tubes were placed on an Eppendorf incubator at 32°C for 30 minutes to allow the sperm to actively leave the epididymes. The tissue residuals were removed from the tubes. The slides were stained and examined by bright field microscope with an oil immersion lens according to published protocol [47]. Two and half thousand sperms per group were scored and the abnormalities were categorized as close as to those described by Wyrobek and Bruce [47]. Abnormal sperms had forms readily recognizable as amorphous, beak, without hook, triangular, banana-shaped, tail abnormality, dwarf, and giant. Sperm count was determined under the light microscope using a Neubauer hematocytometer according to the World Health Organization manual for the examination of human semen [48], and two counts per animal were averaged.

### 4.9. Determination of Oxidative Stress Markers in Sperm Cells

To study the effect of naringin on the oxidative damage induced by diabetes, sperm cells were collected from the remaining caudae epididymes for estimation of lipid peroxidation, GSH, GSSG, and ROS generation. Malonodialdehyde (MDA) generated by lipid peroxidation was quantified in the sperm cells according to the method of Ohkawa et al. [49] based on thiobarbituric acid reactivity. The MDA levels of the samples were calculated from the standard curve using the 1,1,3,3-tetramethoxypropane as the standard and expressed as nmol/mg protein. GSH was assayed with 5,5′-dithiobis(2-nitrobenzoic acid) (DTNB) according to the protocol described by Ellman [50]. GSSG was assayed with DTNB, glutathione reductase, and nicotinamide adenine dinucleotide phosphate as described previously [51]. The concentrations of GSH and GSSG were calculated from standard curves that were obtained from freshly prepared standard solutions of GSH and GSSG, respectively, and expressed as *μ*g/mg protein. The value obtained for GSH was divided by the GSSG value to get the GSH/GSSG ratio. Protein quantitative was carried out by the method of Lowry et al. [52] using bovine serum albumin as a standard.

The generation of intracellular ROS was evaluated based on the intracellular peroxide-dependent oxidation of 2′,7′-dichlorodihydrofluorescein diacetate (DCFH-DA) to form a fluorescent compound, 2′,7′-dichlorofluorescein (DCF) with some modifications as previously described [53]. The sperm cells were collected in tubes containing 1.5 mL fetal calf serum and then centrifuged and washed with ice-cold PBS buffer (pH 7.4). The sperm cells were harvested by centrifugation, washed twice with cold PBS, and finally resuspended in PBS buffer. 200 *μ*L sperm cells (2 × 10^5^) were incubated with 200 *μ*L of DCFH-DA (0.4 *μ*M) for 60 minutes at 37°C in dark. The fluorescence intensity was monitored with a FLUOstar OMEGA microplate reader (BMG LABTECH Ltd., Germany) from an excitation wavelength of 485 nm and an emission wavelength of 520 nm. Results were expressed as fold of nondiabetic control.

### 4.10. Data Analysis

Data were expressed as the mean ± standard deviation (SD) of the means. The data were analyzed by employing nonparametric tests, Mann-Whitney *U* test, or Kruskal-Wallis test followed by Dunn's multiple comparisons test. Results were considered significantly different if the *P* value was less than 0.05.

## Figures and Tables

**Table 1:** DNA strand breaks in bone marrow of nondiabetic and diabetic rats 24 hours after the last treatment with the indicated doses of naringin (4 weeks exposures, spaced 24 hours apart) (mean ± SD).

Treatment groups (mg/kg)	Tail length (*μ*m)	Tail DNA (%)	Tail moment (arbitrary unit)	Olive tail moment (arbitrary units)
Nondiabetic control	14.0 ± 4.8	5.98 ± 1.15	1.96 ± 0.61	4.78 ± 1.23
Nondiabetic + naringin (25)	12.6 ± 4.77	5.76 ± 1.52	2.12 ± 0.43	4.14 ± 0.56
Nondiabetic + naringin (50)	11.8 ± 4.71	4.82 ± 1.76	1.98 ± 0.23	3.20 ± 0.73
Diabetes	27.2 ± 4.91*	14.2 ± 3.39*	4.72 ± 0.54**	9.44 ± 2.03*
Diabetes + naringin (25)	18.6 ± 4.33^a^	8.08 ± 1.54^b^	2.98 ± 0.85^a^	5.82 ± 1.27^b^
Diabetes + naringin (50)	15.8 ± 3.96^b^	6.97 ± 1.53^b^	2.24 ± 0.7^b^	4.32 ± 1.18^b^

*P < 0.05 and **P < 0.01 versus nondiabetic control (Kruskal-Wallis test followed by Dunn's multiple comparisons test). ^a^ *P* < 0.05 and ^b^ *P* < 0.01 versus diabetes alone (Mann-Whitney *U *test).

**Table 2:** Frequencies of micronucleated polychromatic erythrocytes (% MNPCE) and mitotic activity (% PCE) in bone marrow of nondiabetic and diabetic rats 24 hours after the last treatment with the indicated doses of naringin (4 weeks exposures, spaced 24 hours apart) (mean ± SD).

Treatment groups (mg/kg)	% MNPCE (mean ± SD)	% PCE (mean ± SD)
Nondiabetic control	0.32 ± 0.08	48.4 ± 1.81
Nondiabetic + naringin (25)	0.30 ± 0.07	48.2 ± 2.16
Nondiabetic + naringin (50)	0.28 ± 0.08	49.0 ± 1.41
Diabetes	0.92 ± 0.13**	45.6 ± 3.97
Diabetes + naringin (25)	0.60 ± 0.12^a^	47.0 ± 2.54
Diabetes + naringin (50)	0.44 ± 0.11^b^	47.4 ± 2.40

**P < 0.01 versus nondiabetic control (Kruskal-Wallis test followed by Dunn's multiple comparisons test). ^a^ *P* < 0.05 and ^b^ *P* < 0.01 versus diabetes alone (Mann-Whitney *U *test).

**Table 3:** Frequency of spermatocyte chromosomal aberrations in testes of nondiabetic and diabetic rats 24 hours after the last treatment with the indicated doses of naringin (4 weeks exposures, spaced 24 hours apart) (mean ± SD).

Treatment groups (mg/kg)	Different structural chromosomal aberrations screened					Total chromosomal aberrations (%)
	X-Y univalents	Autosomal univalents	F/B	Polyploidy	MV	
Nondiabetic control	7	4	2	0	1	2.8 ± 0.44
Nondiabetic + naringin (25)	6	3	1	—	1	2.4 ± 0.89
Nondiabetic + naringin (50)	5	6	1	0	1	2.6 ± 0.89
Diabetes	22	18	3	2	3	9.60 ± 1.51*
Diabetes + naringin (25)	13	10	2	1	1	5.40 ± 1.81^a^
Diabetes + naringin (50)	6	5	2	2	1	3.20 ± 1.09^b^

*P < 0.05, versus nondiabetic control (Kruskal-Wallis test followed by Dunn's multiple comparisons test). ^a^ *P* < 0.05 and ^b^ *P* < 0.01 versus diabetes alone (Mann-Whitney *U *test). F: fragments; B: breaks; MV: multivalents having a chain of four chromosomes.

**Table 4:** Frequencies of sperm count, abnormalities, ROS, GSSG, GSH, GSSG/GSH ratio, and MDA of nondiabetic and diabetic rats 24 hours after the last treatment with the indicated doses of naringin (4 weeks exposures, spaced 24 hours apart) (mean ± SD).

Treatment groups (mg/kg)	Counts (×10^6^/couda epididymis)	Abnormal sperm (%)	MDA (nmol/mg protein)	GSH (*μ*g/mg protein)	GSSG (*μ*g/mg protein)	GSH/GSSG ratio	ROS (% of control)
Nondiabetic control	46.8 ± 6.7	2.2 ± 0.8	12.6 ± 1.8	1.8 ± 0.4	0.26 ± 0.05	7.4 ± 2.5	100.0 ± 7.4
Nondiabetic + naringin (25)	49.0 ± 5.7	1.8 ± 0.8	11.4 ± 3.2	1.9 ± 0.4	0.24 ± 0.05	8.1 ± 2.0	95.4 ± 8.5
Nondiabetic + naringin (50)	51.0 ± 7.3	1.6 ± 0.54	10.4 ± 2.7	2.2 ± 0.5	0.25 ± 0.07	8.3 ± 2.8	93.2 ± 9.3
Diabetes	34.8 ± 3.3*	6.4 ± 1.3*	19.6 ± 3.7*	0.8 ± 0.2*	0.36 ± 0.04*	2.4 ± 0.8	160.8 ± 15.5*
Diabetes + naringin (25)	41.0 ± 4.2^a^	4.6 ± 0.5^a^	13.6 ± 3.5^b^	1.5 ± 0.18^b^	0.26 ± 0.05^a^	6.0 ± 1.5^b^	125.4 ± 14.9^b^
Diabetes + naringin (50)	46.0 ± 3.6^b^	2.2 ± 0.8^b^	10.8 ± 2.6^b^	1.8 ± 0.15^b^	0.22 ± 0.05^b^	8.5 ± 2.1^b^	106.4 ± 8.7^b^

*P < 0.05 versus nondiabetic control (Kruskal-Wallis test followed by Dunn's multiple comparisons test), ^a^ *P* < 0.05, ^b^ *P* < 0.01 versus diabetes alone (Mann-Whitney *U* test). MDA: malondialdehyde; GSH: reduced glutathione; GSSG: oxidized glutathione; ROS: reactive oxygen species.

## Abbreviations

ROS: Reactive oxygen species
MNPCE: Micronucleated polychromatic erythrocytes
MN: Micronuclei
GSH: Reduced glutathione
GSSG: Oxidized glutathione.
